# Work status among middle-aged and older individuals in China: the effects on physical and mental health

**DOI:** 10.3389/fpubh.2024.1322398

**Published:** 2024-03-28

**Authors:** Yi Fu, Xiaohan Li

**Affiliations:** Business School, Central South University, Changsha, China

**Keywords:** middle-aged and older adults, physical health, mental health, mechanism, work status

## Abstract

**Objective:**

China’s middle-aged and older population is a rich source of human capital. Therefore, considering the health of this group is important when creating and using human resources.

**Methods:**

Using data from the nationwide China Health and Retirement Longitudinal Study (CHARLS) 2018 baseline survey of 19,000 adults ages 45 years and older, this study was an objective investigation of the effects of work on the physical and mental health of middle-aged and older adults. We conducted several types of analyses using details of participants’ lifestyles and demographic characteristics (living environment, education, financial level, and access to medical services) with *work* (i.e., employment or volunteer work) as a primary input component of health production to examine their effects on the health status of middle-aged and older individuals.

**Results:**

Six primary outcomes were identified: (1) Employment positively affects both the physical and mental health of middle-aged and older people; (2) Employment can influence the physical and mental health of middle-aged and older people through income, cognitive level, and social support networks; (3) Compared to non-self-employment, self-employment dramatically worsens physical health but has no apparent detrimental effects on mental health. Compared to non-agricultural employment, agricultural labor affects both physical and mental health. (4) Employment has more positive physical and mental health effects in Individuals with higher rather than lower levels of education. (5) Employment opportunities in the eastern region are more likely to promote better physical health than those in the central and western regions of China, although the effects on mental health are negative. (6) When considering groups at different ages of the lifespan, the 60–65-year-old group, who are just entering retirement probably benefit more from continuing work.

**Conclusion:**

We provide some recommendations to encourage middle-aged and older people to work and utilize their experience, which will enhance their physical and mental well-being and help them in realize their own value and social integration.

## 1 Introduction

The Opinions of the Central Committee of the Communist Party of China and the State Council on Strengthening Work on the older adult in the New Era explicitly emphasize the implementation of a national strategy to actively cope with population aging and focus on considering older adults as an important resource in family, community, and socioeconomic development of the country. This is in response to new demographic development patterns of gradual deepening and accelerating aging in China. As of the end of 2022, 280 million people in China were aged 60 years or older, representing 19.8% of the nation’s total population and represents a significant middle-aged and older demographic dividend that must be capitalized upon. Consequently, encouraging middle-aged and older adults to work and to improve their social engagement has emerged as a prominent objective and comprises a serious issue that require immediate attention. Employment is an important aspect of social participation (1) and a key determinant of health (2). In addition to having rights, middle-aged and older individuals should be able to enjoy a high standard of living and excellent health. Moreover, population aging must be addressed to meet the 2030 sustainable development objectives; hence, researching the link between work and health in middle-aged and older adults is crucial.

## 2 Literature review

Many studies have been conducted on age-related variables influencing health. A key theoretical foundation for the study of health demands has been established according to Grossman’s view of health as a durable good having the triple features of consumer goods, elements of production, and investment goods simultaneously (3). In conclusion, health results from the interaction between multiple micro-, meso- and macro-factors (4). Researchers have begun to analyse the effects of various levels and types of factors on various indicators of subjective self-assessment of health, self-care in daily life, cognitive ability, and mental health, as well as the prevalence of specific diseases in older adults, starting with social and natural factors at the macro-level, community and family factors at the meso-level and biogenetic factors at the micro-level. At the macro- and meso-levels, the primary variables influencing aging health have steadily switched from biological to socioeconomic causes, as chronic and degenerative illnesses have become the main health issues that Chinese older adults face (5). The social level of geriatric healthcare service provision (6), social insurance (7), urban–rural differences (8), medical service utilization (9), socioeconomic status (10), social participation (11), city size (12), family care, and intergenerational support (13) have become the main areas of interest for academics studying the factors affecting aging health. Micro-level research has concentrated on healthy longevity genes to identify and elucidate the relationship between the way of life of older adults, their social and natural environments, and their longevity in their older years (14).

Regarding the effect of work, opinions differ on the significance of social interaction for health. According to various studies, the health of older adults is positively affected by their jobs. Employment prevents diseases brought on by inactivity at home and allows older individuals to continue to enjoy the well-being that comes with employment (15); The ongoing employment of the older population adds to a feeling of work continuity, which keeps them healthy (16); diverse and frequent social activities contribute to improved health and lower mortality rates (17), and retirement may have a long-term detrimental influence on mental health (18). An alternative perspective suggests that older adults’ health may be harmed if they continue to work full-time. Retirement allows more time for social and leisure activities; therefore, quitting the labor market increases life satisfaction for this group (19), and increased sleep and more frequent physical activity after retirement are beneficial for both physical and mental health (20). Additionally, employment analysis has produced inconsistent results. Regarding the nature of the job, self-employment has been shown to benefit older adults’ mental health more than paid employment (21). In terms of age and gender, social participation has a greater promoting effect on the daily activity ability of older women than that of men. Compared to young people, the impact on the health of older adults is greater (22). In terms of health type, employment is also advantageous for general mental health and depression. Due to a dearth of research or contradictory results, data on mortality, physical health, and overall health are limited (23).

In summary, academic studies on the health of middle-aged and older adults have been conducted extensively from a variety of perspectives. Although room for improvement remains, the effect of employment on health has partly caught the attention of academics, offering a valuable reference for this study. First, there are clear discrepancies between the findings of earlier research, with a greater number of studies focusing on mental health and fewer on physical health. Second, there is an overly singular discussion of the nature of work and a lack of systematic research. Third, there are insufficient details regarding the mechanism underlying how employment affects health. Therefore, this paper examines how employment affects the mental and physical health of middle-aged and older populations. It also divides job types into employed and self-employed, agricultural and non-agricultural and examines the effects of each. Finally, the mechanism underlying how employment affects the health of the middle-aged and older population is clarified.

## 3 Methods and hypothesis development

### 3.1 Mechanism analysis

Working can help middle-aged and older people continually realise their worth; this self-affirmation and self-esteem enhancement helps to maintain mental health. Work also provides a regular rhythm of life, which helps to maintain a normal biological clock and has a positive impact on physical health. Thus, Hypothesis 1 is proposed:

*H1:* Employment positively affects both physical and mental health in middle-aged and older adults.

#### 3.1.1 Mechanism related to income

People who work may earn sufficient revenue to cover essential expenses such as housing, food, and medical care. People are more ready and able to invest in their health and pay more attention to it as *per capita* income increases (24). Furthermore, a solid income source may assist individuals in obtaining a better diet and medical treatment, thereby supporting good health. Additionally, having a job increases middle-aged and older people’s incomes and enhances their capacity to help the younger generations, thus raising their social worth (25). Retirement from the workforce often results in lower income and spending, which may exacerbate stress and adversely affect mental health (26). At a certain age, people eventually leave the workforce, and their primary societal function changes from being a ‘source’ of value generation to a ‘burden’ of value consumption. The quality of life of those in the job market is greatly affected by changes in their social roles and lifestyles. The psychology of older adults is affected by changes in their social roles and habits, which increase their vulnerability to anxiety, loneliness, and despair (27). A person’s feelings of identity and self-worth deriving from work may support their psychological health. People gain a sense of their abilities and value through employment, which boosts their self-esteem and contentment. Thus, we propose the following hypothesis:

*H2:* Work may improve the health of middle-aged and older adults by raising their income levels, which in turn can improve their health.

#### 3.1.2 Cognitive level

Employment not only allows ongoing learning and development, but also improves an individual’s cognitive capacity. First, employment allows individuals to be exposed to new information and skills while continuing to learn and enhance their cognitive capacities through practice (28). People’s cognitive abilities are stimulated when they face various obstacles and demands at work, which force them to reflect, decide, and create. By stimulating the brain and physiological processes, such as hormone levels, this cognitive training alters the structure of the brain and lowers the risk of dementia (29). Second, having a job broadens one’s social circle and raises the likelihood of receiving emotional support, both of which improve the ability to adjust to shifts in social development and lower the risk of cognitive problems (30). Additionally, earning a living may improve people’s degree of happiness and well-being in life and lower their risk of cognitive deterioration (31). Higher cognitive function increases an individual’s ability to recognize and comprehend health issues as well as their propensity to make wiser choices and engage in healthy habits. They can comprehend health information more easily, see possibilities and hazards, and make decisions that align with their health requirements. They may also be more likely to adopt health-promoting habits that can improve their physical health, such as eating well, exercising often, and listening to doctors. Furthermore, those with greater cognitive abilities are better able to recognize and accept their feelings and frustrations and develop healthy coping mechanisms to manage stress and challenges. They have a better understanding of their inner desires, emotions, and personality traits. This helps individuals evaluate their skills, values, and sense of self-worth more accurately, leading to a lower tendency toward depression (32, 33). Thus, we propose the following hypothesis:

*H3:* Employment may enhance the health of middle-aged and older adults through increased cognitive function.

#### 3.1.3 Social support network

Less social support may increase the likelihood of social isolation in middle-aged and older adults, which can have detrimental effects on both physical and mental health (34). Work may boost opportunities for face-to-face contact (35), widen social communication channels, boost social support, meet emotional needs, and reduce loneliness, all of which may reduce depression (36). In addition, social participation can reduce the risk of physical dysfunction in middle-aged and older persons (37). In general, marital ties, intimate relationships, and individual organizational engagement are the three basic components of social support networks (38). First, employment could make it easier to participate in associations, training, public charity, and labor union-related events. Second, employment can increase the likelihood of receiving intergenerational support and feedback from adult children and forming resource-sharing and win-win situations (39). Finally, seeking job opportunities to collaborate and communicate with friends increases the frequency of interaction with them. Increasing economic and emotional interactions with children, as well as social interactions with friends, can help improve the mental health of middle-aged and older adults (40, 41). Research has been shown that these exchanges may also greatly enhance their physical well-being (17). Thus, the following hypothesis is proposed:

*H4:* By expanding the reach of social support networks, employment enhances the physical and mental well-being of middle-aged and older adults.

### 3.2 Model construction

#### 3.2.1 Basic model construction

This paper’s health production function is grounded in Grossman’s (3) idea that a variety of input parameters including lifestyle, living environment, education, financial level, and access to medical services, generate health. This study treats work as a primary input component of health production and examines how it affects the health status of middle-aged and older individuals.

(1) $$H=f\left(x\right)=f\left(\mathrm{working},\;H1,H2.\text{.}\right)$$

In Equation (1), H represents the health level. The variable ‘working’ represents employment status, which is the key input factor for health production analysed in this research. H1 and H2 represent other factors affecting the level of health.

This study verifies the effects of employment on physical and mental health separately using the following regression models:

(2) self:health=α0+α1working+γX+ε

(3) cesd10p=α0+α1working+γX+ε

In Equations (2), (3), self_health represents self-assessed health, which is used to measure the physical health of middle-aged and older people; cesd10p represents the depression level of older people; ‘working’ represents employment status, X represents other control variables, and ε represents the residuals.

#### 3.2.2 Influence mechanism test

To confirm the transmission mechanism of employment’s impact on middle-aged and older individuals’ health, and to investigate the potential mediating role of income mechanism, cognitive ability, and social support network in this relationship, this study is structured into two steps for model construction:

In the first step, the regression model of the impact of employment status on the mediating variables of personal income (lincomeind), cognitive level (lcognition), and social support network (social_network) is constructed, with the specific model being set in Equations (4)–(6):

(4) lincomeind=β0+β1working+λX+μ

(5) lcognition=β0+β1working+λX+μ

(6) social:network=β0+β1working+λX+μ

In the second step, assuming that the regression test in the first step is passed, the three mediating variables of personal income, cognitive level, and social support network are added as control variables to the regression model of employment status on health, and the changes in regression coefficients are used to determine whether there is a mediating effect in the mechanism of the influence of employment status on health, with the specific model being set in Equations (7)–(12):

(7) self:health=γ0+γ1working+γ2lincomeind+θX+ν

(8) self:health=γ0+γ1working+γ2lcognition+θX+ν

(9) self:health=γ0γ1working+γ2social:network+θX+ν

(10) cesd10p=γ0γ1working+γ2lincomeind+θX+ν

(11) cesd10p=γ0+γ1working+γ2lcognition+θX+ν

(12) cesd10p=γ0+γ1workingγ2social:network+θX+ν

#### 3.2.3 Variable selection

The two primary explanatory factors are mental and physical health. Self-assessed health is often used to gage physical health (42). Moreover, the CESD10 is often used to assess mental health (43). To facilitate the interpretation of the findings, both indicators, which are negatively coded in the CHARLS data,—are positively coded in this work.

The employment status variable is the primary explanatory factor; a value of 0 indicates *never having been employed*, 1 indicates *having previously worked but not presently working*, and 2 indicates *currently working*.

The social support system, cognitive ability, and personal income of the respondents are the mediating factors. Personal income is measured by revenue earned in the previous year, including wage income, transfer income, and old-age insurance/pension. The sum of the scores from the Simple Mental Evaluation, Word Recall, Minus-7 Numerical Operations, and Delayed Recall assessments indicates an individual’s cognitive level; higher scores indicate a better cognitive level, which is a positive indicator. Interactions with friends, financial interactions with children, and organizational participation are indicators of social support networks.

The control variables in this study include the household macroenvironment and demographic features in the pertinent literature. Gender, education level, age, health insurance coverage, and place of residence are examples of these demographic traits. Interactions with spouses and children are part of the family environment. The primary components of the macroenvironment are the medical level, air quality, and age-appropriate renovations. For the specific variable settings, see Table 1.

**Table 1:** Variable settings, definitions, and measurements.

Variable name	Definitions	Measurements
Self_health	Self-assessed health	1 for very poor, 2 for poor, 3 for fair, 4 for good, 5 for very good
cesd10p	Mental health	The original CESD10 negative indicator is reversed and assigned a value range of 0–3, and the positive indicator value range is also set to 0–3.
Working	Employment status	1 for worked but is unemployed right now 2 for working, and 0 for never worked.
Age	Age	Year of interview minus year of birth
Edu	Educational level	Ten values ranging from 1 to 10, representing illiteracy to a master’s degree.
Rural	Urban and rural areas	1 for urban, 0 for rural
Gender	Gender	1 for male, 0 for female
Childconnection	Frequency of contact with children	From 1 to 10, it means the frequency is getting lower and lower
Married	Spouse	1 for having a spouse, 0 for not having one
Lperincome	*Per capita* household income	The logarithm of each family member’s income plus one. From the previous year divided by all family
Self_healthself_health	Medical satisfaction	A score of 1 to 5 indicates increased dissatisfaction with the local health services’ quality, affordability, and accessibility.
Air_quality	Air quality satisfaction	The current year’s satisfaction score for air quality ranges from 1 to 5, which indicates a rise in the degree of dissatisfaction
Handicapped_Facilities	Barrier-free access	1 for yes, 2 for no
Lfood	*Per capita* household food expenditure	The logarithm of food expenses plus one over the previous week divided by the total number of people in the home
Insurance	Medical insurance	0 for no health insurance, 1 for any kind of health insurance
Region2	Central region dummy variables	/
Region3	Eastern region dummy variable	/
Lincomeind	Personal income	The logarithm of all forms of income (transfer, salary, old-age insurance, and pension) plus one for the previous year
lcognition	Cognitive level	Total points for the word recall, delayed recollection, subtracting seven numerical operations, and summary mental assessment plus one logarithmic calculation.
Social_network1	Friends interaction	Record visiting and interacting with friends as 1 and providing help to friends as 1, and add the two indicators together
Social_network2	Children’s economic interactions	Allocate one unit for each of the children’s and respondent’s two-way economic contacts, and then sum them up.
Social_network3	Organizational participation	Participation in public service and community group activities should be totalled as one each.

### 3.3 Data sources

For this study, we used data from the China Health and Retirement Longitudinal Study (CHARLS) 2018 national baseline survey of adults ages 45 and above in 150 counties and 450 communities or villages in 28 Chinese provinces (including municipalities directly under the central government and autonomous regions). The survey included 12,400 households and 19,000 respondents overall. The CHARLS questionnaire covers a variety of topics including basic personal information, health status, employment, retirement and pensions, family income, consumption, assets, and fundamental community data. These data were used to examine China’s aging population, encourage multidisciplinary studies, and provide a stronger scientific foundation for the development and enhancement of relevant Chinese policies. Table 2 presents the variables’ descriptive statistics.

**Table 2:** Descriptive statistics.

Variable	Obs	Value	Mean/%	Std. dev
Self_health	11,325	1	5.21% 20.37% 50.31% 12.49% 11.62%	1
		2		
		3		
		4		
		5		
cesd10p	11,325	0–30	20.508	7.045
Working	11,325	0	19%	0.456
		1	28.19%	
		2	71.63%	
Age	11,325	45–108	61.86	9.805
Married	11,325	0	20.68%	0.405
		1	79.32%	
Edu	11,325	1	22.83%	1.893
		2	21.63%	
		3	0.19%	
		4	22.62%	
		5	21.32%	
		6	7.61%	
		7	2.08%	
		8	1.03%	
		9	0.66%	
		10	0.01%	
Rural	11,325	0	84.03%	0.366
		1	15.97%	
Gender	11,325	0	52.83%	0.499
		1	47.17%	
childconnection	11,325	1	10.2%	2.588
		2	15.72%	
		3	20.41%	
		4	14.72%	
		5	15.16%	
		6	4.81%	
		7	2.26%	
		8	1.01%	
		9	11.77%	
		10	3.95%	
sat_health_care	11,325	1	15.56%	1.09
		2	22.93%	
		3	45.32%	
		4	7.51%	
		5	8.68%	
lperincome	11,325	0–14.914	6.215	3.712
medinsurance	11,325	0	2.68%	0.161
		1	97.32%	
lfood	11,325	0–8.74	4.366	1.378
Air_quality	11,325	1	4.71%	0.828
		2	27.57%	
		3	51.72%	
		4	12.90%	
		5	3.11%	
Handicapped_facilities	11,325	1	10.45%	0.617
		2	89.55%	
lincomeind	11,325	0–15.607	5.938	4.119
lcognition	11,325	0–3.289	2.720	0.894
Social_network1	11,325	0	61.09%	0.663
		1	29.46%	
		2	9.46%	
Social_network2	11,325	0	8.36%	0.510
		1	51.29%	
		2	40.34%	
Social_network3	11,325	0	95.26%	0.224
		1	2.98%	
		2	1.76%	

## 4 Results and discussion of empirical investigation and test analyses

### 4.1 Benchmark regression analysis

In the benchmark regressions, factors including demographics, family environment, and macroenvironment are controlled for using self-assessed health and CESD10p as explanatory variables and job status as the core explanatory variable. Table 3 displays the results of ordinary least squares (OLS) regressions with and without the control variables (Models (1) and (2) and Models (3) and (4), respectively). According to the regression analysis, work has a stronger beneficial impact on mental than on physical health, with coefficient effects on self-assessed and mental health being considerably favorable. As China’s economy grows, more middle-aged and older individuals pursue the inherent value of having a job (44); thus, the psychological health effect of employment is more prominent. The regression results for the control variables were generally consistent with expectations; however, there were differences in the effects of certain variables on physical and mental health. The impact of having a partner on mental health is significantly positive, and the impact on physical health is also positive, but not significant, which is consistent with previous research results (45). This is because having a partner can improve respondents’ psychological well-being by providing greater mental comfort. Health insurance has a significant negative effect on both mental and physical health. Although the coverage rate of China’s health insurance is high, the utilization rate is low, the proportion of out-of-pocket medical expenses is still high, and there are many reimbursement thresholds that prevent it from playing its due role (46); The effect of age-friendly modifications on health is insignificant, which may be because there are only 1,184 age-friendly modifications in this sample, which does not form a scale effect to produce a significant effect.

**Table 3:** Benchmark regression results.

	(1)	(2)	(3)	(4)
Variables	self_health	cesd10p	self_health	cesd10p
Working	0.237***	1.045***	0.185***	0.535***
	(11.30)	(6.92)	(7.96)	(3.34)
Age			−0.011***	−0.050***
			(−9.88)	(−6.50)
Married			0.011	1.277***
			(0.49)	(7.85)
Edu			0.040***	0.571***
			(7.30)	(15.22)
Rural			0.210***	1.441***
			(7.80)	(8.26)
Gender			0.111***	1.914***
			(5.68)	(14.45)
childconnection			−0.013***	−0.231***
			(−3.32)	(−8.62)
sat_health_care			−0.118***	−0.629***
			(−13.06)	(−10.48)
lperincome			0.011***	0.148***
			(4.30)	(8.48)
medinsurance			−0.133**	−0.093
			(−2.33)	(−0.23)
lfood			0.011	0.117**
			(1.60)	(2.39)
Air_quality			−0.134***	−0.988***
			(−11.39)	(−12.29)
Handicapped_facilities			−0.002	0.023
			(−0.12)	(0.23)
Region2			0.155***	1.294***
			(7.21)	(8.39)
Region3			0.348***	2.310***
			(14.72)	(14.16)
Constant	2.643***	18.717***	3.772***	21.474***
	(70.64)	(68.66)	(29.65)	(24.30)
Observations	11,325	11,325	11,325	11,325
*R*-squared	0.012	0.005	0.098	0.160

*p < 0.1, **p < 0.05, ***p < 0.01. Standard errors are reported in parentheses.

### 4.2 Robustness tests

#### 4.2.1 Replacing explanatory variables

This paper uses activities of daily living (ADL) to replace self-assessment of health. ADL is assessed by asking respondents whether there are difficulties in six daily activities such as eating, dressing, and bathing. Each item is scored using a 4-point scale (no difficulty = 1, difficult but can be completed = 2, difficult to complete even with help = 3, cannot be completed = 4), and the sum of the six scores is the composite score of the ability of older adults to perform daily activities, which is a negative indicator. Using health satisfaction instead of CESD10p, scores from 1 to 5 represent a gradual increase in the degree of dissatisfaction; this indicator is also negative. The regression findings in Table 4 (1, 2) remain significant, even when the explanatory variables are changed.

**Table 4:** Robustness test.

	(1)	(2)	(3)	(4)	(5)	(6)
Variables	adl	sat_health	self_health	cesd10p	self_health	cesd10p
working	−0.838***	−0.169***	0.381***	0.117***	0.293***	1.019***
	(−11.12)	(−7.87)	(8.47)	(2.83)	(15.71)	(7.91)
Control variables	YES	YES	YES	YES	YES	YES
Observations	11,325	11,325	11,325	11,325	14,492	14,492
*R*-squared	0.180	0.115			0.091	0.146

*p < 0.1, **p < 0.05, ***p < 0.01. Standard errors are reported in parentheses.

#### 4.2.2 Replacing the regression method

This study used ordered logit regression to examine the robustness of the findings, as self-assessed health is an ordered categorical variable. The regression results are presented in Table 4 (3, 4). Results show that after changing the model, the findings remain valid.

#### 4.2.3 Replacement of data

The harmonized CHARLS dataset is created using baseline survey data collected in 2011, 2013, 2015, and 2018. The dataset was cleaned and compiled into panel data, with temporal and individual effects considered for the regression. Regression findings are shown in Table 4 (5, 6), where they remain significant.

### 4.3 Endogeneity issues

This study investigates the effects of work on health. Nonetheless, a person’s health may have a direct or indirect influence on their capacity and performance at work, which in turn affects employment. To guarantee that the analyses were unaffected by health status and address the endogeneity issue, those who had never worked or had changed occupation because of health issues were excluded from the sample selection procedure. To lessen the potential consequences of endogeneity, an instrumental variable, employment status from the previous round, was used with a one-period lag of the explanatory factors (47). The regression results are presented in Table 5; (1) shows the one-stage regression results and (2, 3) show the two-stage regression results. These findings support the conclusions of previous studies. Another instrumental variable is the average employment status of the community. On the one hand, this instrumental variable represents the employment status of residents in the region. People are often influenced by those around them, especially family, friends, and neighbors, which may lead to a convergence of behavior within the group. Therefore, the instrumental variable is positively correlated with the respondents’ employment status, which meets the correlation requirements of the instrumental variable; On the other hand, the employment status of others is not related to the respondents’ personal status and meets the requirements of exogeneity. Table 5 displays the regression findings. The one-stage regression findings are shown in (4), and the two-stage regression results are shown in (5, 6). The impact is still trending positively, supporting the previous conclusion.

**Table 5:** Endogeneity Test I.

	(1)	(2)	(3)	(4)	(5)	(6)
Variables	Working	self_health	cesd10p	Working	self_health	cesd10p
Working		0.452***	0.951***		1.045***	7.655***
		(10.64)	(3.21)		(2.90)	(3.21)
Working2015	0.526***					
	(47.32)					
iv				0.136***		
				(5.97)		
Control variables	YES	YES	YES	YES	YES	YES
Observations	10,662	10,662	10,662	11,325	11,325	11,325
*R*-squared	0.482	0.084	0.160	0.243	0.106	0.306

*p < 0.1, **p < 0.05, ***p < 0.01. Standard errors are reported in parentheses.

Furthermore, considering the potential for bidirectional causation between the independent and dependent variables, the joint equations were estimated using the three-stage least squares (3SLS) approach; 3SLS is widely regarded as a more effective parameter estimation technique that yields more accurate estimation results because it not only addresses the issue of endogenous explanatory variables but also considers the relationships between perturbation terms in various equations (48). The results of the regression analysis are presented in Table 6 (1–4). The findings support the preceding findings, in part, because they demonstrate that work has a considerable impact on both physical and mental health. However, the effects of either variable on employment are not statistically significant.

**Table 6:** Endogeneity Test II.

	(1)	(2)	(3)	(4)	(5)	(6)
Variables	self_health	Working	cesd10p	Working	self_health	cesd10p
Working	0.451***		0.948***		0.190***	0.557***
	(10.84)		(3.37)		(8.37)	(3.60)
self_health		0.027				
		(1.56)				
cesd10p				0.002		
				(0.68)		
Control variables	YES	YES	YES	YES	YES	YES
Observations	11,325	11,325	11,325	11,325	11,322	11,322
*R*-squared	0.084	0.479	0.160	0.480	0.098	0.160

*p < 0.1, **p < 0.05, ***p < 0.01. Standard errors are reported in parentheses.

Given that this empirical study excluded samples that had never worked or switched jobs because of health problems, this may have led to sample self-selection problems. Therefore, propensity score matching was used to match sample data and eliminate the effects of confounding factors. The stability of our findings is further demonstrated by the regression results after kernel matching, which are displayed in Table 6 (5, 6), and are consistent with the basic regression results.

### 4.4 Mechanism test

The findings shown in Table 7 indicate that work has a considerable positive impact on personal income, and that personal income also acts as a moderator in the relationship between employment and the mental and physical health of middle-aged and older adults. First, people’s physical health considerably increases with an increase in money. People spend more money on wholesome foods such as whole grains, fresh produce, fresh fruit, and high-quality protein sources. The body receives the nutrients it needs from a well-balanced diet, which boosts immunity and lowers the risk of chronic illnesses. Higher wages may be used to pay for more comprehensive health insurance, medical supplies, and wellness programs, as well as regular physicals, immunisations, health check-ups, and prescription drugs. Maintaining general physical health and preventing and detecting health issues early is made possible by routine examinations and prompt treatment. Second, a higher salary has a major positive impact on an individual’s mental health. Higher income provides more resources to access mental health support, including counseling, psychotherapy, or other mental health services. In addition, individuals have greater flexibility and opportunities to improve their work-life balance and increase personal satisfaction and well-being.

**Table 7:** Income mechanism test.

Variables	(1)	(2)	(3)
	lincomeind	self_health	cesd10p
Working	0.455***	0.181***	0.490***
	(8.48)	(7.77)	(3.04)
lincomeind		0.011***	0.124***
		(4.39)	(7.77)
Control variables	YES	YES	YES
Observations	11,325	11,325	11,325
*R*-squared	0.720	0.098	0.159

*p < 0.1, **p < 0.05, ***p < 0.01. Standard errors are reported in parentheses.

According to the data in Table 8, employment has a substantial impact on the cognitive level, mediates the impact of work on mental health, and has no significant influence on the impact of employment on physical health. Increased cognitive function considerably improves the health of middle-aged and older adults. Individuals with higher cognitive capacity are often better at solving problems and making decisions. They have improved problem-solving and understanding skills as well as the ability to respond appropriately. This skill lessens worry and sadness and improves their capacity to handle obstacles in life and at work. Furthermore, individuals with strong cognitive abilities often have more adaptable, optimistic, and proactive mindsets. They often adopt optimistic, logical, and adaptable perspectives when understanding and reacting to life experiences and events. They are more adept at manageing and restraining their cognitive processes to prevent pessimism and excessive concern, which supports their mental wellness.

**Table 8:** Cognitive level mechanism test.

Variables	(1)	(2)	(3)
	lcognition	self_health	cesd10p
working	0.052***	0.184***	0.460***
	(2.88)	(7.94)	(2.92)
lcognition		0.009	1.441***
		(0.71)	(15.23)
Control variables	YES	YES	YES
Observations	11,325	11,325	11,325
*R*-squared	0.341	0.098	0.182

*p < 0.1, **p < 0.05, ***p < 0.01. Standard errors are reported in parentheses.

The analyses of friend contact, economic engagement with children, and social organizational involvement are shown in Tables 9–11 respectively. The regression analysis show that employment can improve social interaction with friends, children, and social organization participation. These factors can broaden an individual’s social sphere, strengthen the stability of social networks, and provide access to up-to-date medical and healthcare information, all of which can improve physical health. Conversely, engaging in social services and public welfare endeavors may enhance physical well-being through physical activity and enhancing physical functioning within appropriate limits. Furthermore, middle-aged and older populations experience less loneliness due to these social activities, and their mental health may be further enhanced by the happiness and self-affirmation they get from them.

**Table 9:** Mechanistic tests of friend interactions.

Variables	(1)	(2)	(3)
	social_network1	self_health	cesd10p
Working	0.054***	0.181***	0.519***
	(3.77)	(7.80)	(3.24)
Social_network1		0.076***	0.292***
		(5.56)	(3.28)
Control variables	YES	YES	YES
Observations	11,325	11,325	11,325
*R*-squared	0.038	0.100	0.162

*p < 0.1, **p < 0.05, ***p < 0.01. Standard errors are reported in parentheses.

**Table 10:** Mechanistic tests of interaction with the child’s economy.

Variables	(1)	(2)	(3)
	social_network2	self_health	cesd10p
Working	0.087***	0.183***	0.517***
	(6.12)	(7.86)	(3.22)
Social_network2		0.024*	0.210**
		(1.66)	(2.11)
Observations	11,325	11,325	11,325
*R*-squared	0.024	0.098	0.161

*p < 0.1, **p < 0.05, ***p < 0.01. Standard errors are reported in parentheses.

**Table 11:** Social organization participation mechanisms test.

Variables	(1)	(2)	(3)
	social_network3	self_health	cesd10p
Working	0.012**	0.183***	0.529***
	(2.30)	(7.90)	(3.30)
Social_network3		0.132***	0.510***
		(3.81)	(2.62)
Control variables	YES	YES	YES
Observations	11,325	11,325	11,325
*R*-squared	0.036	0.099	0.161

*p < 0.1, **p < 0.05, ***p < 0.01. Standard errors are reported in parentheses.

### 4.5 Heterogeneity analyses

#### 4.5.1 Job type heterogeneity

The work settings, demands, and labor intensities associated with various job types have varying effects on psychological and physical health. Consequently, the types of work in the CHARLS are categorized in this study. Self-employed and non-self-employed work types are categorized in Table 12(1, 2), where self-employment is given a value of 1 and non-self-employment is assigned a value of 0. According to the regression results, self-employment significantly lowers a person’s physical well-being, which is consistent with Cheng et al.’s findings (49). This could be partially because independent contractors sometimes shoulder more risks and obligations; they also must cope with a wide range of work-related duties, management issues, and decision-making. This high level of stress may result in lengthy workdays and limited time for relaxation, both of which could be harmful to an individual’s physical well-being. Self-employment also has a negative but non-significant effect on mental health (50).

**Table 12:** Heterogeneity of job types.

Variables	(1)	(2)	(3)	(4)
	self_health	cesd10p	self_health	cesd10p
working	0.270***	0.647***	0.267***	0.951***
	(9.57)	(3.41)	(8.37)	(4.63)
working*worktype1	−0.064***	−0.084		
	(−5.28)	(−1.04)		
working*worktype2			−0.054***	−0.272***
			(−3.82)	(−3.07)
Control variables	YES	YES	YES	YES
Observations	11,325	11,325	11,325	11,325
*R*-squared	0.100	0.160	0.099	0.161

*p < 0.1, **p < 0.05, ***p < 0.01. Standard errors are reported in parentheses.

The types of work are divided into agriculture and non-agriculture, as shown in Table 12 (3, 4). Agriculture-related employment is scored as 1, while non-agricultural employment as 0. The regression results show that working in agriculture negatively affects both physical and mental health. Agricultural work typically involves considerable physical labor, including tasks such as plowing, harvesting, pruning, and transportation. This can be challenging for middle-aged and older people, whose health has declined, and can easily lead to physical discomfort, such as fatigue, muscle pain, and joint problems. People working in agriculture spend considerable time outside, where they are exposed to chemicals, pesticides, farm equipment, and high-risk conditions for plants and animals. These factors can lead to physical health problems. In addition, the stress and uncertainty associated with agricultural work, such as natural disasters, market fluctuations, and economic pressure, may cause psychological stress and anxiety in middle-aged and older adults. This, in turn, adversely affects the physical and mental health of middle- and older adults.

#### 4.5.2 Education level heterogeneity

Studies have found that people with higher education levels are more likely to remain employed (51, 52). As a result, the sample for this study is split into two groups according to educational attainment: those with high school education and beyond (scored as 1) and those with less education (scored as 0). Table 13 (1, 2) present the regression results. Highly educated people perceive greater benefits to their physical and mental health as a beneficial consequence of their jobs. They often have access to better workplaces and professional prospects. Engaging in less physically demanding and more stable jobs reduces physical load and work stress, positively affecting mental and physical health.

**Table 13:** Education levels and regional heterogeneity.

Variables	(1)	(2)	(3)	(4)
	self_health	cesd10p	self_health	cesd10p
working	0.167***	0.321**	0.161***	0.537***
	(7.16)	(1.99)	(6.01)	(2.84)
working*edu	0.090***	0.730***		
	(5.60)	(7.44)		
working*region			0.075*	−0.006
			(1.70)	(−0.02)
Control variables	YES	YES	YES	YES
Observations	11,325	11,325	11,325	11,325
*R*-squared	0.096	0.146	0.098	0.160

*p < 0.1, **p < 0.05, ***p < 0.01. Standard errors are reported in parentheses.

#### 4.5.3 Regional heterogeneity

Considering the different levels of economic development in East, Central, and West China, there are significant differences in the employment opportunities offered. Therefore, this study divides the region into east and central-west, with the eastern region assigned a value of 1 and the central-west assigned a value of 0. Table 13 (3, 4) present the regression results. Employment opportunities in the eastern region can contribute to the improvement of individuals’ physical health, while the effect on mental health is negative, although not significant. This may be because the eastern regions, which are more developed, have certain advantages in protecting the rights and interests of workers, contributing to the improvement of physical health. However, these regions often face intense competition and poor working environments. Individuals are often exposed to great work, competition, and time pressures, which may lead to anxiety, depression, and other mental health problems.

#### 4.5.4 Age heterogeneity

With the increasing life expectancy, population aging has become an important feature of our society now and for a long time to come. Population aging has a profound impact on economic and social operations. Therefore, it is important to explore the health of individuals of different age groups. In this paper, the respondents were divided into two age groups. Table 10 (1, 2) divide the age group into 45–60 and 60–65 years, with 60–65 years assigned a value of 1, and 45–60 years assigned a value of 0. The regression results clearly show that employment has a more positive impact on health in the 60–65 age group. For middle-aged and older age groups, the transition from permanent working status to retirement may have an impact on health. Retirement may lead to social isolation, reduced physical activity, and a lack of meaning and purpose, which may affect physical and mental health. Therefore, for the 60–65-year-old group who are just in retirement, continued employment may have a more positive effect. Table 14 (3, 4) divide the age group into 60–65 and 65+ years, with 65+ assigned a value of 1 and 60–65 assigned a value of 0. Although employment no longer has a positive effect on physical health in older age groups, it can contribute to psychological wellbeing by providing cognitive stimulation and social connectedness.

**Table 14:** Age heterogeneity.

Variables	(1)	(2)	(3)	(4)
	self_health	cesd10p	self_health	cesd10p
working	0.122***	0.346	0.255***	0.465**
	(3.70)	(1.59)	(7.90)	(2.05)
working*age1	0.073***	0.268*		
	(3.30)	(1.80)		
working*age2			−0.039**	0.375***
			(−2.05)	(2.81)
Control variables	YES	YES	YES	YES
Observations	7,253	7,253	6,372	6,372
*R*-squared	0.103	0.146	0.079	0.162

*p < 0.1, **p < 0.05, ***p < 0.01. Standard errors are reported in parentheses.

## 5 Conclusions and policy recommendations

China’s current middle-aged and older population has abundant human resources. When developing and utilizing human resources, the health of this group should not be neglected. Using data from the CHARLS2018 baseline survey, this study objectively investigates the effects of work on the physical and mental health of middle-aged and older adults. The following outcomes were found: (1) middle-aged and older adults who work have better physical and mental health; and (2) employment influences middle-aged and older adults’ income, cognitive function, and social support systems, all of which affect their physical and mental health. (3) Compared with non-self-employment, self-employment dramatically worsens physical health but has no apparent detrimental effects on mental health. Compared to non-agricultural employment, agricultural labor is harmful to both physical and mental health; (4) Those with higher education benefit more from employment in terms of both physical and mental health; (5) Employment opportunities in the Eastern region are more likely to promote improved physical health but negatively affect mental health than those in the Midwest; (6) Examining different age groups reveals that individuals in the 60–65-year-old group, who are only recently retired or about to retire, are likely to experience a more positive effect from continued employment. Hence, the following suggestions are recommended.

Initially, middle-aged and older should be aggressively encouraged to work, considering that using this labor force may be a significant step toward the advancement of aging careers, and thorough preparations should be made to support aging adults in the workplace. Appropriate government agencies should encourage re-employment for older adults with a positive view of aging, which should modify their conceptions and methods of governance when developing policies on employment for older adults.

Second, given the detrimental effects of self-employment and agricultural labor on health, more middle-aged and older individuals may benefit from actively participate in their social livesn flexible and diverse ways, such as part-time jobs, short-term contracts, and teleworking. To guarantee that middle-aged and older adults have a reliable source of income and comfortable living circumstances, they must be provided with full social care and welfare support, including housing assistance, medical protection, and pension insurance.

Finally, to fully realise the health benefits of hiring low-age retirees, we must intensify our efforts to combat age discrimination and enforce existing laws and regulations to guarantee that middle-aged and older individuals are not subjected to discrimination or treated unfairly during the employment process. To eliminate the prejudice associated with aging on the side of employers and society at large, we also need to raise awareness of the value and experiences of middle-aged and older individuals.

By taking these steps, middle-aged and older people can be encouraged to work, make the most of their skills and experiences, and provide them with full welfare assistance, all of which will contribute to the creation of a welcoming and peaceful workplace. This will enhance the physical and mental well-being of middle-aged and older adults while assisting them in realizing their own value and social integration.

## Data availability statement

Publicly available datasets were analyzed in this study. This data can be found at: https://charls.pku.edu.cn/.

## Author contributions

YF: Conceptualization, Funding acquisition, Supervision, Writing – review & editing. XL: Data curation, Software, Writing – original draft.

## References

[1] Anger WK Elliot DL Bodner T Olson R Rohlman DS Truxillo DM. Effectiveness of total worker health interventions. J Occup Health Psychol. (2015) 20:226–47. doi: 10.1037/a0038340, PMID: 25528687

[2] Rözer JJ Hofstra B Brashears ME Volker B. Does unemployment lead to isolation? The consequences of unemployment for social networks. Soc Netw. (2020) 63:100–11. doi: 10.1016/j.socnet.2020.06.002, PMID: 19250999

[3] Grossman M. On the concept of health capital and the demand for health. J Pol Econ. (1972) 80:223–55. doi: 10.1086/259880, PMID: 38439067

[4] Hood CM Gennuso KP Swain GR Catlin BB. County health rankings relationships between determinant factors and health outcomes. Am J Prev Med. (2016) 50:129–35. doi: 10.1016/j.amepre.2015.08.024, PMID: 26526164

[5] Daniel H Bornstein SS Kane GC Hlth Public Policy Comm Amer C. Addressing social determinants to improve patient care and promote health equity: an American College of Physicians position paper. Ann Intern Med. (2018) 168:577. doi: 10.7326/M17-2441, PMID: 29677265

[6] Kruk ME Gage AD Joseph NT Danaei G García-Saisó S Salomon JA. Mortality due to low-quality health systems in the universal health coverage era: a systematic analysis of amenable deaths in 137 countries. Lancet. (2018) 392:2203–12. doi: 10.1016/S0140-6736(18)31668-4, PMID: 30195398 PMC6238021

[7] Yuan H Chen SQ Pan GC Zheng LY. Social pension scheme and health inequality: evidence from China’s new rural social pension scheme. Front Public Health. (2021) 9:837431. doi: 10.3389/fpubh.2021.837431, PMID: 35198537 PMC8858825

[8] Singh GK Siahpush M. Widening rural-urban disparities in life expectancy, US, 1969–2009. Am J Prev Med. (2014) 46:E19–29. doi: 10.1016/j.amepre.2013.10.017, PMID: 24439358

[9] Liao PA Chang HH Yang FA. Does the universal health insurance program affect urban-rural differences in health service utilization among the elderly? Evidence from a longitudinal study in Taiwan. J Rural Health. (2012) 28:84–91. doi: 10.1111/j.1748-0361.2011.00363.x, PMID: 22236318

[10] Meyer OL Castro-Schilo L Aguilar-Gaxiola S. Determinants of mental health and self-rated health: a model of socioeconomic status, neighborhood safety, and physical activity. Am J Public Health. (2014) 104:1734–41. doi: 10.2105/AJPH.2014.302003, PMID: 25033151 PMC4151927

[11] Croezen S Avendano M Burdorf A van Lenthe FJ. Social participation and depression in old age: a fixed-effects analysis in 10 European countries. Am J Epidemiol. (2015) 182:168–76. doi: 10.1093/aje/kwv015, PMID: 26025236 PMC4493978

[12] Chen J Davis DS Wu K HJ DH. Life satisfaction in urbanizing China: the effect of city size and pathways to urban residency. Cities. (2015) 49:88–97. doi: 10.1016/j.cities.2015.07.011

[13] Xu HW. Physical and mental health of Chinese grandparents caring for grandchildren and great-grandparents. Soc Sci Med. (2019) 229:106–16. doi: 10.1016/j.socscimed.2018.05.047, PMID: 29866373 PMC6261790

[14] Zeng Y Feng QS Gu DN Vaupel JW. Demographics, phenotypic health characteristics and genetic analysis of centenarians in China. Mech Ageing Dev. (2017) 165:86–97. doi: 10.1016/j.mad.2016.12.010, PMID: 28040447 PMC5489367

[15] Santos ACS Ribeiro BGA Martins JT Galdino MJQ Robazzi MLDCC Ribeiro RP. Motivations of retired professors to return to work activities at a public university. Rev Rene. (2016) 17:561–8. doi: 10.15253/2175-6783.2016000400017

[16] Lichtenthaler PW Fischbach A. Job crafting and motivation to continue working beyond retirement age. Career Dev Int. (2016) 21:477–97. doi: 10.1108/CDI-01-2016-0009

[17] Krokstad S Ding D Grunseit AC Sund ER Holmen TL Rangul V. Multiple lifestyle behaviours and mortality, findings from a large population-based Norwegian cohort study – the HUNT study. BMC Public Health. (2017) 17:58. doi: 10.1186/s12889-016-3993-x, PMID: 28068991 PMC5223537

[18] Heller-Sahlgren G. Retirement blues. J Health Econ. (2017) 54:66–78. doi: 10.1016/j.jhealeco.2017.03.007, PMID: 28505541

[19] Zhang AQ Zhang Y Tao YW. Does retirement make people happier?-evidence from China. Front Public Health. (2022) 10:874500. doi: 10.3389/fpubh.2022.874500, PMID: 35784195 PMC9247314

[20] Eibich P. Understanding the effect of retirement on health: mechanisms and heterogeneity. J Health Econ. (2015) 43:1–12. doi: 10.1016/j.jhealeco.2015.05.001, PMID: 26079117

[21] Peng J Jincai Z Maria VLA Dwumfour OC Juan L. Does participation in local non-agricultural employment improve the mental health of elderly adults in rural areas? Evidence from China. Front Public Health. (2021) 9:746580. doi: 10.3389/fpubh.2021.746580, PMID: 34778181 PMC8578795

[22] Tomioka K Kurumatani N Hosoi H. Age and gender differences in the association between social participation and instrumental activities of daily living among community-dwelling elderly. BMC Geriatr. (2017) 17:99. doi: 10.1186/s12877-017-0491-7, PMID: 28454521 PMC5410028

[23] van der Noordt M Ijzelenberg H Droomers M Proper KI. Health effects of employment: a systematic review of prospective studies. Occup Environ Med. (2014) 71:730–6. doi: 10.1136/oemed-2013-101891, PMID: 24556535

[24] Chetty R Stepner M Abraham S Lin S Scuderi B Turner N. The association between income and life expectancy in the United States, 2001–2014. JAMA. (2016) 315:1750–66. doi: 10.1001/jama.2016.4226, PMID: 27063997 PMC4866586

[25] Tian SL Xu L Wu XL. Impacts of social participation on self-rated health of aging women in China: with a mediating role of caring for grandchildren. Int J Environ Res Public Health. (2021) 18:5790. doi: 10.3390/ijerph18115790, PMID: 34071221 PMC8198177

[26] Cheng Y Lan J Ci Q. Employment and mental health of the chinese elderly: Evidence from CHARLS 2018. Int. J. Environ. Res. Public Health. (2023) 20:2791. doi: 10.3390/ijerph20042791 36833488 PMC9956944

[27] Vo K Forder PM Tavener M Rodgers B Banks E Bauman A. Retirement, age, gender and mental health: findings from the 45 and up study. Aging Ment Health. (2015) 19:647–57. doi: 10.1080/13607863.2014.962002, PMID: 25271125

[28] Cai S. Does social participation improve cognitive abilities of the elderly? J Popul Econ. (2022) 35:591–619. doi: 10.1007/s00148-020-00817-y, PMID: 38388406

[29] Shen C Rolls ET Cheng W Kang J Dong G Xie C. Associations of social isolation and loneliness with later dementia. Neurology. (2022) 99:e164–75. doi: 10.1212/WNL.0000000000200583, PMID: 35676089

[30] Fan Z Lv X Tu L Zhang M Yu X Wang H. Reduced social activities and networks, but not social support, are associated with cognitive decline among older Chinese adults: a prospective study. Soc Sci Med. (2021) 289:114423. doi: 10.1016/j.socscimed.2021.114423, PMID: 34597879

[31] Mosca I Wright RE. Effect of retirement on cognition: evidence from the Irish marriage bar. Demography. (2018) 55:1317–41. doi: 10.1007/s13524-018-0682-7, PMID: 29881982 PMC6060984

[32] Zhou LN Ma XC Wang W. Relationship between cognitive performance and depressive symptoms in Chinese older adults: the China health and retirement longitudinal study (CHARLS). J Affect Disord. (2021) 281:454–8. doi: 10.1016/j.jad.2020.12.059, PMID: 33360747

[33] Chen YJ Wang K Zhao JX Zhang ZX Wang JY He L. Overage labor, intergenerational financial support, and depression among older rural residents: evidence from China. Front Public Health. (2023) 11:1219703. doi: 10.3389/fpubh.2023.1219703, PMID: 37680270 PMC10482248

[34] Webster NJ Ajrouch KJ Antonucci TC. Volunteering and health: the role of social network change. Soc Sci Med. (2021) 285:114274. doi: 10.1016/j.socscimed.2021.114274, PMID: 34390978 PMC8416937

[35] Fingerman KL Ng YT Zhang SY Britt K Colera G Birditt KS. Living alone during COVID-19: social contact and emotional well-being among older adults. J Gerontol B Psychol Sci Soc Sci. (2021) 76:E116–21. doi: 10.1093/geronb/gbaa200, PMID: 33196815 PMC7717423

[36] Mackenzie CS Abdulrazaq S. Social engagement mediates the relationship between participation in social activities and psychological distress among older adults. Aging Ment Health. (2021) 25:299–305. doi: 10.1080/13607863.2019.1697200, PMID: 31818117

[37] Kanamori S Kai Y Aida J Kondo K Kawachi I Hirai H. Social participation and the prevention of functional disability in older Japanese: the JAGES cohort study. PLoS One. (2014) 9:e99638. doi: 10.1371/journal.pone.0099638, PMID: 24923270 PMC4055714

[38] Berkman LF Syme SL. Social networks, host resistance, and mortality: a nine-year follow-up study of Alameda County residents. Am J Epidemiol. (2017) 185:1070–88. doi: 10.1093/aje/kwx103, PMID: 30052734

[39] Zhang XJ Chen W. Does grandchild care intention, intergenerational support have an impact on the health of older adults in China? A quantitative study of CFPS data. Front Public Health. (2023) 11:1186798. doi: 10.3389/fpubh.2023.1186798, PMID: 37693722 PMC10484214

[40] Buber I Engelhardt H. Children’s impact on the mental health of their older mothers and fathers: findings from the survey of health, ageing and retirement in Europe. Eur J Ageing. (2008) 5:31–45. doi: 10.1007/s10433-008-0074-8, PMID: 28798560 PMC5546383

[41] Chen YX Feeley TH. Social support, social strain, loneliness, and well-being among older adults: an analysis of the health and retirement study. J Soc Personal Relat. (2014) 31:141–61. doi: 10.1177/0265407513488728

[42] Freedman VA Kasper JD. Cohort profile: the national health and aging trends study (NHATS). Int J Epidemiol. (2019) 48:1044–1045g. doi: 10.1093/ije/dyz109, PMID: 31237935 PMC6934030

[43] Mohebbi M Nguyen V McNeil JJ Woods RL Nelson MR Shah RC. Psychometric properties of a short form of the Center for Epidemiologic Studies Depression (CES-D-10) scale for screening depressive symptoms in healthy community dwelling older adults. Gen Hosp Psychiatry. (2018) 51:118–25. doi: 10.1016/j.genhosppsych.2017.08.002, PMID: 28890280 PMC6178798

[44] Zhou DS Zhan QQ Li LL. The impact of self-employment on mental health of the younger elderly in China. BMC Geriatr. (2023) 23:280. doi: 10.1186/s12877-023-03948-5, PMID: 37158843 PMC10166046

[45] Myroniuk TW. Marital dissolutions and the health of older individuals in a rural African context. J Gerontol B Psychol Sci Soc Sci. (2017) 72:656–64. doi: 10.1093/geronb/gbw077, PMID: 27382043 PMC5927165

[46] Wang Y Jiang Y Li Y Wang XJ Ma C Ma SG. Health insurance utilization and its impact: observations from the middle-aged and elderly in China. PLoS One. (2013) 8:e80978. doi: 10.1371/journal.pone.0080978, PMID: 24324654 PMC3855696

[47] Chang H Ding Q Zhao W Hou N Liu W. The digital economy, industrial structure upgrading, and carbon emission intensity —— empirical evidence from China’s provinces. Energ Strat Rev. (2023) 50:101218. doi: 10.1016/j.esr.2023.101218

[48] Adewuyi AO Awodumi OB. Biomass energy consumption, economic growth and carbon emissions: Fresh evidence from West Africa using a simultaneous equation model. Energy. (2017) 119:453–471. doi: 10.1016/j.energy.2016.12.059

[49] Cheng MW Zhang YS. The impact of self-employment on population health – evidence from the China health and nutrition survey. J Guangxi Univ. (2022) 44:115–25. doi: 10.13624/j.cnki.jgupss.2022.01.021

[50] Ahn T. Employment and health among older people: self-employment vs. wage employment. Appl. Econ. Lett. (2020) 27:1574–1580. doi: 10.1080/13504851.2019.1697795

[51] Pettersson J. Instead of bowling alone? Unretirement of pensioners in Sweden. Int J Manpow. (2014) 35:1016–37. doi: 10.1108/IJM-09-2012-0136

[52] Pleau RL. Gender differences in postretirement employment. Res Aging. (2010) 32:267–303. doi: 10.1177/0164027509357706, PMID: 37462123

